# Supplementary material for: Bioinformatic Analysis of the Leptin–Ob-R Interface: Structural Modeling, Thermodynamic Profiling, and Stability in Diverse Microenvironments
Source: Int J Mol Sci. 2025 Jul 20;26(14):6955. doi: 10.3390/ijms26146955 (PMC12295314; doi:10.3390/ijms26146955)
Supplement: Supplementary file 1 [file ijms-26-06955-s001.zip › ijms-3715096_Table S1.docx]

**Table S1.** Intermolecular interactions^a^ between amino acids in the docking interfaces of five mammalian leptin–CRH2 complexes at 36.5 and 37.5 °C.

| **Heterodimer leptin–CRH2 model at 37.5 °C** | | | | | |
| --- | --- | --- | --- | --- | --- |
| **Human** (51) | | **Mouse** (59) | **Rat** (57) | **Pig** (53) | **Macaque** (49) |
| Y441/Q75 | | D615/K5 | Y439/R20 | L506/N82 | R613/D9 |
| P561/T19 | | F502/E81 | Y470/D9 | F563/T19 | L503/86 |
| L506/T10 | | F502/D85 | R467/L89 | S507/D85 | F502/E81 |
| L506/L86 | | F502/L86 | R613/T12 | L505/N82 | I501/N82 |
| S507/V89 | | L440/N82 | L504/L89 | S470/D85 | S505/L89 |
| Y472/K5 | | L440/Q75 | Y470/F92 | L506/D9 | P559/T19 |
| L471/F92 | | Y470/D9 | E563/K15 | V562/K15 | F561/T12 |
| F563/T12 | | L503/L13 | L440/H78 | L506/V6 | P562/T16 |
| F563/T16 | | V560/D23 | P559/T19 | S507/L89 | L440/N82 |
| L471/V89 | | F561/T16 | S468/F92 | R615/D9 | L503/N82 |
| Y441/R20 | | Y470/K5 | V560/T16 | S507/L86 | L504/D9 |
| S470/S93 | | L469/F92 | V560/R20 | S470/L89 | L469/L89 |
| L506/V6 | | L536/R20 | Y439/I76 | F563/T12 | Y470/D9 |
| L442/D79 | | L504/V6 | L504/D9 | L506/L89 | S505/D85 |
| I503/N82 | | R467/F92 | S468/S93 | Y472/L89 | L440/N78 |
| V562/T19 | | I501/N82 | F502/N82 | L506/L86 | S467/L89 |
| P564/T12 | | P562/T16 | L469/L89 | F504/N82 | V560/K15 |
| P564/T16 | | S468/S93 | R466/D85 | G440/R20 | L504/L86 |
| Y472/F92 | | Y470/L89 | L504/L13 | L442/D79 | H439/R20 |
| L506/N82 | | F502/N82 | F561/T16 | Y472/D9 | S468/F82 |
| Y472/V89 | | V560/T19 | D530/D9 | V562/R20 | F502/N82 |
| R615/T12 | | Y470/V1 | L504/T10 | L471/L89 | S468/S83 |
| V562/K15 | | R613/T12 | P562/K15 | Y472/V6 | R466/D85 |
| L442/N78 | | E563/L15 | P562/T16 | P564/T16 | V560/R20 |
| Y472/V1 | | Y439/D79 | L504/L86 | F504/D85 | L504/V6 |
| L505/N82 | | R466/D85 | L440/D79 | R615/T12 | Y470/V1 |
| L506/D9 | | R613/D9 | Y470/V6 | I503/N82 | Y470/K5 |
| L506/L13 | | S468/L89 | S505/L89 | L506/L13 | Y470/V6 |
| F504/N82 | | G438/R20 | V560/D23 | S470/S92 | S468/H88 |
| D617/Y5 | | Y439/I79 | Y470/K5 | L538/R20 | D530/D9 |
| F504/D85 | | P472/V1 | F561/T12 | Y472/R5 | F561/T16 |
| S470/F92 | | L504/L89 | R467/F92 | L505/L13 | D615/K5 |
| L505/L13 | | D530/D9 | S468/D85 | R468/D85 | P562/T12 |
| S470/V89 | | P562/K15 | Y439/Q75 | V562/T16 | S505/L86 |
| P564/K15 | | S505/D85 | P562/T12 | L442/N82 | V560/T16 |
| Y472/D9 | | L440/N78 | D615/K5 | Y472/S92 | L503/L13 |
| V562/D23 | | Y439/Q96 | Y439/D79 | F563/T16 | L504/T19 |
| V562/R20 | | L503/N82 | G438/R20 | Y441/R20 | L440/D79 |
| Y472/V6 | | S505/L89 | S505/D85 | D532/D9 | L469/F92 |
| S469/F92 | | S468/H88 | L503/L13 | D617/R5 | S468/D85 |
| Y441/D79 | | V560/T16 | F502/D85 | S469/L89 | L504/L89 |
| S469/V89 | | L504/D9 | R613/D9 | Y472/V1 | P562/K15 |
| F504/E81 | | L503/L86 | L440/N82 | Y441/Q75 | F472/V1 |
| G618/K5 | | F561/T12 | S505/L86 | S470/H88 | Y470/L89 |
| L506/V89 | | V560/R20 | Y470/V1 | L442/N78 | V560/T19 |
| D532/D9 | | Y470/V27 | S468/L89 | L471/S92 | F502/D85 |
| S39/H88 | | L504/T10 | T441/H78 | G508/L89 | G506/L89 |
| L442/N82 | | L504/L86 | L469/F92 | V562/T19 | S468/L89 |
| S470/D85 | | L469/L89 | L503/L86 | L506/T10 | R613/T12 |
| R615/D9 | | P562/T12 | V560/K15 | P564/K15 |  |
| V562/T16 | | R467/L89 | F502/E81 | P564/T12 |  |
|  | | P559/T19 | S468/H88 | V562/D23 |  |
|  |  | Y439/R20 | I501/N82 | F504/E81 |  |
|  |  | S505/L86 | V560/T19 |  |  |
|  |  | V560/K15 | L503/N82 |  |  |
|  |  | S468/F92 | Y470/L89 |  |  |
|  |  | F561/T19 | I501/H78 |  |  |
|  |  | L440/D79 |  |  |  |
|  |  | L504/N82 |  |  |  |
| **Heterodimer leptin–CRH2 model at 36.5 °C** | | | | | |
| **Human** (51) | | **Mouse** (59) | **Rat** (57) | **Pig** (53) | **Macaque** (49) |
| F563/T12 | Y470/V1 | Y470/L89 | S470/S92 | S468/H88 |  |
| S470/V89 | L503/L12 | I501/I78 | L505/N82 | F502/F85 |  |
| P561/T19 | R613/D9 | P559/T19 | V562/T16 | D615/K5 |  |
| Y441/Q75 | L469/F92 | Y470/V1 | S507/D85 | L503/L86 |  |
| L506/V89 | P562/T12 | S505/L89 | L442/D79 | S505/D85 |  |
| L506/L86 | L536/R20 | R613/D9 | F563/T19 | S468/D85 |  |
| P564/T12 | F561/T12 | L503/N82 | P564/K15 | V560/T19 |  |
| R615/D9 | L503/N82 | S468/F92 | Y472/V6 | L440/N78 |  |
| Y441/D79 | L469/L89 | Y439/R20 | G440/R20 | L440/D79 |  |
| S470/S93 | I501/N82 | L469/F92 | S470/H88 | Y470/K5 |  |
| L506/V6 | F502/G81 | V560/D23 | Y472/S92 | L503/N82 |  |
| D617/K5 | L504/T10 | P562/Y15 | F504/N82 | F472/V1 |  |
| L506/N82 | L504/L86 | F561/T33 | R615/T12 | S467/L89 |  |
| V562/R20 | V560/T19 | L504/T10 | Y472/V1 | L504/L86 |  |
| Y472/V6 | L440/N82 | Y439/Q75 | V562/D23 | P562/K15 |  |
| R615/T12 | L504/D9 | F502/E81 | L506/L13 | F561/T16 |  |
| V562/K15 | S468/S93 | Y470/F92 | L506/V6 | S468/S93 |  |
| S39/H88 | F561/T19 | S468/H88 | L506/T10 | S505/S86 |  |
| I503/N82 | Y570/D9 | F502/N82 | L442/N78 | L469/F92 |  |
| L442/N82 | L504/V6 | L503/L13 | F504/E81 | Y470/V1 |  |
| L506/D9 | Y470/V6 | V560/T19 | D617/R5 | L469/L89 |  |
| L505/L17 | Y439/D79 | R466/D85 | L471/S92 | F502/E81 |  |
| L442/D79 | S505/L86 | S468/D85 | Y441/R20 | V560/R20 |  |
| P504/N82 | D530/D9 | V560/R20 | L505/L13 | F561/T12 |  |
| V562/T16 | V560/K15 | Y470/V6 | F504/D85 | S468/F92 |  |
| Y472/D9 | S468/F92 | D530/D9 | V562/T19 | D530/D9 |  |
| S470/D85 | R613/T12 | V560/Y15 | P564/T16 | H439/R20 |  |
| L506/L13 | Y439/I76 | L504/L86 | R615/D9 | L504/T10 |  |
| Y472/F92 | V560/T16 | I501/N103 | Y441/N75 | S468/L89 |  |
| L505/N82 | S468/L89 | P562/T16 | S470/L89 | P559/T19 |  |
| F563/T16 | L440/N78 | R467/L89 | Y472/D9 | G506/L89 |  |
| F504/E81 | F502/L86 | S505/D85 | L506/L89 | V560/K15 |  |
| P564/K15 | L440/D79 | R613/T12 | R468/D85 | R613/T12 |  |
| L506/V89 | S505/D85 | L440/N82 | L471/L89 | L504/D9 |  |
| S469/F92 | P472/V1 | S505/L86 | S469/L89 | V560/T16 |  |
| Y472/V89 | Y439/R20 | L504/D9 | S507/L86 | P562/T16 |  |
| P564/T16 | S468/H88 | Y470/D9 | V562/K15 | Y470/D9 |  |
| Y572/V1 | F502/D85 | S468/S93 | F563/T16 | L504/V6 |  |
| S469/V89 | F502/N82 | D615/K5 | L506/L86 | I501/N82 |  |
| V562/D23 | L504/N82 | Y439/D79 | I503/N82 | Y470/V6 |  |
| L506/T10 | P559/T19 | L440/H78 | Y472/L89 | L504/L89 |  |
| D532/D9 | L440/Q75 | F502/D85 | S470/D85 | Y470/L89 |  |
| G618/K5 | V560/D23 | P562/T12 | Y472/R5 | F502/N82 |  |
| Y441/R20 | G438/R20 | E563/K15 | L506/D9 | L503/L13 |  |
| S507/V89 | V560/R20 | Y439/I76 | F563/T12 | R466/D85 |  |
| F504/D85 | Y470/K5 | Y470/K5 | V562/R20 | S505/L89 |  |
| V562/T19 | P562/K15 | L440/D79 | D532/D9 | P562/T12 |  |
| S470/F92 | D615/K5 | R467/F92 | G508/L89 | R613/D9 |  |
| L442/N78 | S505/L89 | S468/L89 | L442/N82 | L440/N103 |  |
| Y472/K5 | P562/T16 | L469/L89 | L506/N82 |  |  |
| L471/F92 | R467/F92 | L503/L86 | S507/L89 |  |  |
|  | F561/T16 | V560/T16 | L538/R20 |  |  |
|  | L503/L86 | G438/R20 | P564/T12 |  |  |
|  | E563/K15 | T441/H78 |  |  |  |
|  | R467/L89 | L504/L13 |  |  |  |
|  | R504/D85 | F561/T16 |  |  |  |
|  | L504/L89 | L504/L89 |  |  |  |
|  | Y439/Q75 |  |  |  |  |
|  | Y470/L89 |  |  |  |  |
| ^a^These data were obtained in PRODIGY at 37.5 °C, 36.5 °C and 5.5 Å cut-off distances. The number in parentheses indicates the total number of interactions. In this table, the numbering of the residues is given based on each biological species. In all columns, the first residue corresponds to CRH2 and the second to leptin. | | | | | |
|  |  |  |  |  |  |
