# Supplementary material for: Bioinformatic Analysis of the Leptin–Ob-R Interface: Structural Modeling, Thermodynamic Profiling, and Stability in Diverse Microenvironments
Source: Int J Mol Sci. 2025 Jul 20;26(14):6955. doi: 10.3390/ijms26146955 (PMC12295314; doi:10.3390/ijms26146955)
Supplement: Supplementary file 1 [file ijms-26-06955-s001.zip › Supplementary Material.docx]

Supplementary Material

1. File S1. Coordinates of the Protein–Complex Molecular Dynamics Model (PCMDM) <https://doi.org/10.1080/07391102.2022.2029568>
2. Table S1 lists the intermolecular interactions at the docking interfaces of the five models analyzed here. At 36.5 °C and 37.5 °C, we found 51, 59, 57, 53, and 49 interactions in the human, mouse, rat, pig, and macaque complex models, respectively.
